# Statistical Characteristics of Stationary Flow of Substance in a Network Channel Containing Arbitrary Number of Arms

**DOI:** 10.3390/e22050553

**Published:** 2020-05-15

**Authors:** Roumen Borisov, Zlatinka I. Dimitrova, Nikolay K. Vitanov

**Affiliations:** 1 Institute of Mechanics, Bulgarian Academy of Sciences, Acad. G. Bonchev Str., Bl. 4, 1113 Sofia, Bulgaria; r.borisov@outlook.com; 2 Georgi Nadjakov Institute of Solid State Physics, Bulgarian Academy of Sciences, Blvd. Tzarigradsko Chaussee 72, 1784 Sofia, Bulgaria; zdim@issp.bas.bg; 3 Max-Planck Institute for the Physics of Complex Systems, Noethnitzerstr. 38, 01187 Dresden, Germany

**Keywords:** network, flow, channel, probability distribution, Shannon information measure

## Abstract

We study flow of substance in a channel of network which consists of nodes of network and edges which connect these nodes and form ways for motion of substance. The channel can have arbitrary number of arms and each arm can contain arbitrary number of nodes. The flow of substance is modeled by a system of ordinary differential equations. We discuss first a model for a channel which arms contain infinite number of nodes each. For stationary regime of motion of substance in such a channel we obtain probability distributions connected to distribution of substance in any of channel’s arms and in entire channel. Obtained distributions are not discussed by other authors and can be connected to Waring distribution. Next, we discuss a model for flow of substance in a channel which arms contain finite number of nodes each. We obtain probability distributions connected to distribution of substance in the nodes of the channel for stationary regime of flow of substance. These distributions are also new and we calculate corresponding information measure and Shannon information measure for studied kind of flow of substance.

## 1. Introduction

Complex systems have been studied intensively in the last decades as such systems are encountered frequently in the area natural sciences, population dynamics, social sciences, etc. [1,2,3,4,5,6,7,8,9,10,11]. Large number of phenomena in above systems can be studied by network models [12,13,14,15,16]. One class of phenomena is connected to certain kinds of motion of substance through channels of networks (such as, e.g., migration flows, logistic flows, transport of substances, etc.) [17,18,19,20,21,22,23,24,25]. In the last years we have studied several cases of flow of substance in channels of networks [23,26,27,28,29,30,31]. At the beginning, our studies have been inspired by application of studied models to channels of human migration. One direction of this research is related to probability distributions connected to motion of substance in studied channel of a network. At the beginning we have used model for channel with single arm. After that we have obtained results for channels containing two or three arms. Below we extend this theory for flow of substance in a class of channels containing arbitrary number of arms. We consider the situation where studied channel has a main arm which is the root for other arms. There are special nodes in this channel: the nodes where split of an arm happens. We shall consider the case where more than one arm can arise by this split.

The study presented below is carried out from point of view of possible application of discussed model to various practical cases. Because of this we use the general terms substance, channel and nodes of a network. The model can be applied to different kinds of substances and for flows of these substances in any channel which can be modeled by chains of nodes of a network. We note that the probability distributions obtained below are not discussed by other authors and these distributions can be connected to interesting long-tail distributions (e.g., to the Waring distribution) for particular case where the channel contains single arm.

The organization of text is as follows. In Section 2 we discuss a model of channel containing arms consisting of infinite number of nodes in each of them. We obtain probability distributions connected to amounts of substance in nodes of the arms of this channel for case of stationary flow of substance. Then we study the case of stationary flow of substance in channel which arms contain finite number of nodes each. In Section 3 we discuss the information measure and the Shannon information measure connected to the obtained probability distributions. Short discussion is presented in Section 4 and several concluding remarks are summarized in Section 5.

## 2. Results

### 2.1. Flow of Substance in a Channel Consisting of Arms Containing Infinite Number of Nodes Each

**Figure 1:**
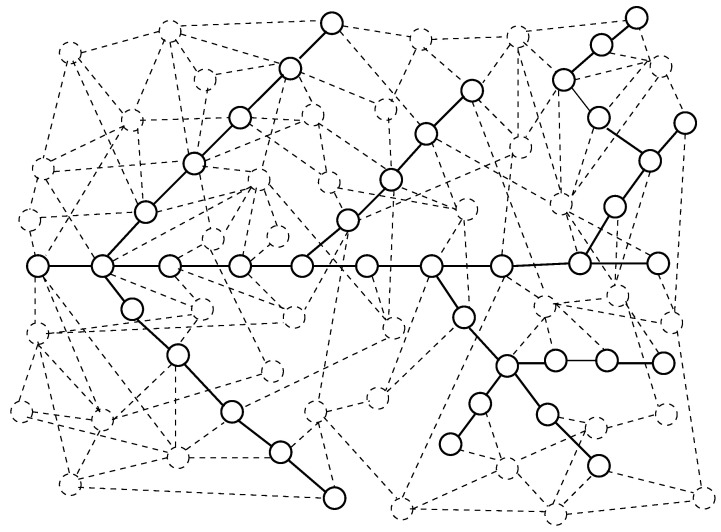
Network and studied channel. Nodes and edges which belong to the channel are marked by solid lines. Other nodes and edges of the network are marked by dashed lines.

The model of motion of substance through the channel discussed below is an extension of model discussed in [23,24]. The channel consists of chains of nodes of a network—Figure 1.

**Figure 2:**
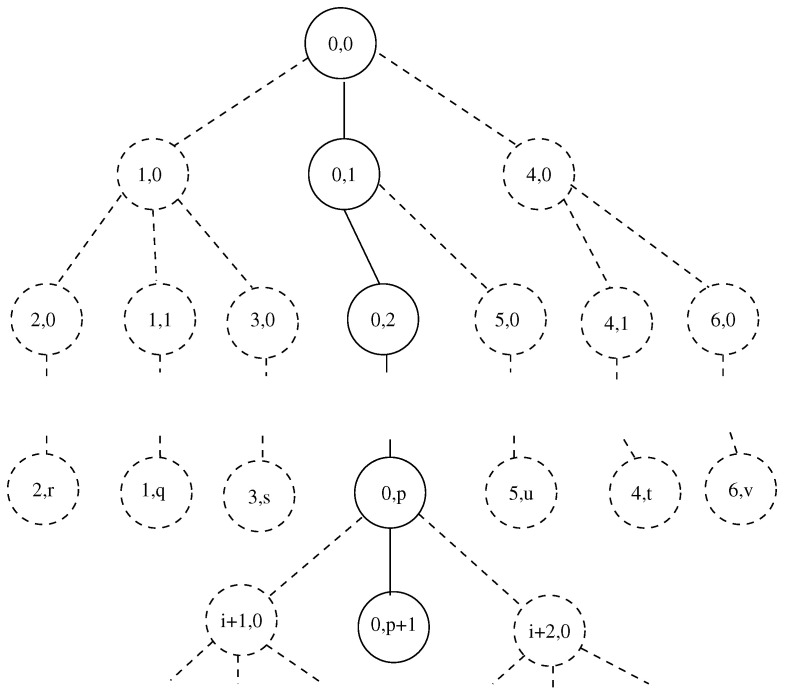
The channel and numbering of its nodes. Only the lower two indexes of numbering of nodes are shown.

The convention for numbering of nodes of channel is as follows—Figure 2. Let us denote a node of the channel by N. We associate 4 indexes to each node: $N_{i,j}^{a,b}$. The lower indexes specify position of node in current arm. *i* is the number associated with current arm. *j* is the the number of node of *i*-th arm. Upper indexes specify the origin of arm *i*. The index *a* is number of arm from which arm *i* splits. The index *b* is number of node of arm *a* where this split happens. Thus a=3,b=8 means: arm *i* of the channel arises from node 8 of arm 3. Then $N_{3,5}^{1,2}$ means: 5-th node of arm 3 which splits at node 2 of channel’s arm 1.

We assume that some substance can enter studied channel from external environment only through the 0-th node of main arm of the channel (this arm is labeled by q=0 below in the text). In addition the substance can move only in one direction in any of arms (from nodes labeled by smaller values of index *j* to nodes labeled by larger values of index *j*). Nodes of each arm are connected by edges and each node is connected only to two neighboring nodes of the arm except for special nodes where a split of an arm happens. These special nodes can be connected to 1 or more additional nodes. In addition we assume that substance can quit the nodes of channel and can move to environment. This process will be called “leakage”. As substance can enter the channel only through 0-th node of main arm then leakage is possible only in direction from channel nodes to other network nodes (and not in the opposite direction).

We stress the following. The node where arm *i* begins is labeled as 0-th node of *i*-th arm. This node is the next one after splitting at node *b* of arm *a*. Thus any of nodes of the channel has unique notation. This is illustrated in Figure 2. The 0-th node of arm 1 arises from 0-th node of the arm 0 (the 0-th node of arm 0 is “environment” which supplies substance to 0-th node of arm 1).

We can consider each node as a cell (box), in other words, we consider the arm to be an array of infinite number of cells indexed in succession by non-negative integers. We assume that an amount $x_{q}^{a_{q},b_{q}}$ of some substance is distributed among cells of the arm *q* which splits at node ($a_{q},b_{q}$) of the network. This substance can move from one cell to another cell.

Let $x_{q,i}^{a_{q},b_{q}}$ be amount of substance in *i*-th cell of the *q*-th arm of the channel. We consider in this section a channel containing infinite number of nodes in each of its arms. Then

(1) $$x_{q}^{a_{q},b_{q}}=\sum_{i=0}^{\infty }x_{q,i}^{a_{q},b_{q}}.$$

The fractions $y_{q,i}^{a_{q},b_{q}}=x_{q,i}^{a_{q},b_{q}}/x_{q}^{a_{q},b_{q}}$ can be considered as probability values of distribution of a discrete random variable $\zeta_{q}$ in corresponding arm of channel

(2) $$y_{q,i}^{a_{q},b_{q}}=p_{q}^{a_{q},b_{q}}\left(\zeta_{q}=i\right),\;i=0,1,\ldots$$

We can define another distribution: the distribution of substance in entire channel. The total amount of substance in this case is

(3) $$x=\sum_{q=0}^{M}x_{q}^{a_{q},b_{q}}=\sum_{q=0}^{M}\sum_{i=0}^{\infty }x_{q,i}^{a_{q},b_{q}},$$

where M+1 is number of arms of channel (remember the main arm of channel which has number 0). Corresponding distribution of substance in the channel is

(4) $$z_{q,i}^{a_{q},b_{q}}=\frac{x_{q,i}^{a_{q},b_{q}}}{x}$$

Next we assume that the amount $x_{q,i}^{a_{q},b_{q}}$ of substance in *i*-th node of *q*-th arm of channel can change because of following processes:

1. Some amount $s_{q}^{a,b}$ of substance enters arm *q* from external environment through 0-th cell of corresponding arm. We consider two kinds of external environments for an arm of the channel
  (a) For the root of the channel (arm with label q=0): substance $s_{0}^{0,0}$ enters the root through environment of the channel
  (b) For the arms of the channel which are not root (i.e., which number is q≠0): Substance $s_{q}^{a,b}$ is part of substance presented in node (a,b) of parent arm. This substance “leaks” from the parent arm to corresponding child arm.
  The substance $s_{q}^{a,b}$ is presented only in node 0 of *q*-th arm of channel. For other nodes of channel there is no substance which enters the node from environment of channel.
2. Amount $f_{q,i}^{a,b}$ from $x_{q,i}^{a,b}$ is transferred from the *i*-th cell to (i+1)-th cell of *q*-th arm;
3. Amount $g_{q,i}^{a,b}$ of $x_{q,i}^{a,b}$ leaks out *i*-th cell of *q*-th arm to environment of the arm of channel. This leakage can be of two kinds
  (a) Leakage to the environment of channel: this kind of leakage leads to loss of substance for the channel
  (b) Leakage to other arms of the channel which begin from the node *b* of the arm *a*: This leakage is connected to the substance $s_{q}^{a,b}$ which enters corresponding child arm of channel which splits from node *b* of arm *a*.

We assume that the process of motion of substance is continuous in time. Then the motion of substance among nodes of *q*-th arm can be modeled mathematically by a system of ordinary differential equations:

(5) $$\begin{array}{ccc} \frac{dx_{q,0}^{a,b}}{dt} & = & s_{q}^{a,b}-f_{q,0}^{a,b}-g_{q,0}^{a,b};\\ \frac{dx_{i,q}^{a,b}}{dt} & = & f_{q,i-1}^{a,b}-f_{q,i}^{a,b}-g_{q,i}^{a,b},\;i=1,2,\ldots ,; \end{array}$$

There are two regimes of work of the channel: stationary regime and non-stationary regime. We shall discuss below the stationary regime of work. In this regime $dx_{i,q}^{a,b}/dt=0$, i=0,1,…. Let us mark the quantities for the stationary regime with ${}^{*}$. Then from (5) one obtains

(6) $$f_{q,0}^{*a,b}=s_{q}^{*a,b}-g_{q,0}^{*a,b};\;\;f_{q,i}^{*a,b}=f_{q,i-1}^{*a,b}-g_{q,i}^{*a,b}$$

We assume the following relationships for amounts of moving substances in (5) ($\alpha_{i},\beta_{i},\gamma_{i},\sigma_{0}$ are parameters):

(7) $$\begin{array}{ccc} s_{0}^{0,0} & = & \sigma_{0}x_{0,0}^{0,0}>0\\ s_{q}^{a,b} & = & \delta_{q}x_{a,b}^{c,d};\;\;1\geq \delta_{q}\geq 0\\ f_{q,i}^{a,b} & = & \left(\alpha_{q,i}^{a,b}+i\beta_{q,i}^{a,b}\right)x_{q,i}^{a,b};\;\;\;1>\alpha_{q,i}^{a,b}>0,\;1\geq \beta_{q,i}^{a,b}\geq 0\\ g_{q,i}^{a,b} & = & {\hat{\gamma }}_{q,i}^{a,b}x_{q,i}^{a,b};\;\;\;1\geq {\hat{\gamma }}_{q,i}^{a,b}\geq 0\to \mathrm{non}-\mathrm{uniform}\\ & & \mathrm{leakage}\;\mathrm{in}\;\mathrm{the}\;\mathrm{nodes}. \end{array}$$

Indexes *c* and *d* in the second of above relationships describe parent arm (numbered by *c*) and parent node of the arm *c* (numbered by *d*) for the arm *q*. $\beta_{q,i}^{a,b}$ accounts for circumstances which lead substance to leave faster the node *i*. ${\hat{\gamma }}_{q,i}^{a,b}$ is a quantity specific for the present study. ${\hat{\gamma }}_{q,i}^{a,b}=\gamma_{q,i}^{a,b}+\sum_{p\in (q,i)}\delta_{p,q,i}^{a,b}$ describes the situation with leakages in cells. $\gamma_{q,i}^{a,b}$ is the leakage to environment from *i*-th node of *q*-th arm. $\delta_{p,q,i}^{a,b}$ describes the leakage to the nodes which split from *i*-th node of *q*-th arm. The notation p∈(q,i) in the sum means all arms which arise from node *i* of arm *q*.

On the basis of all above the model system of differential equations for *q*-th arm of channel becomes

(8) $$\begin{array}{ccc} \frac{dx_{q,0}^{a,b}}{dt} & = & s_{q}^{a,b}-\alpha_{q,0}^{a,b}x_{q,0}^{a,b}-{\hat{\gamma }}_{q,0}^{a,b}x_{q,0}^{a,b};\\ \frac{dx_{q,i}^{a,b}}{dt} & = & \left[\alpha_{q,i-1}^{a,b}+\left(i-1\right)\beta_{q,i-1}^{a,b}\right]x_{q,i-1}^{a,b}-\left(\alpha_{q,i}^{a,b}+i\beta_{q,i}^{a,b}+{\hat{\gamma }}_{q,i}^{a,b}\right)x_{q,i}^{a,b};\;\;i=1,2,\ldots \end{array}$$

Below we shall discuss the situation in which a stationary state exists in entire channel. Then we have $dx_{0}^{q}/dt=0$ in first of the Equations (8). Hence

(9) $$x_{q,0}^{*a,b}=\frac{s_{q}^{a,b}}{\alpha_{0,q}^{a,b}+{\hat{\gamma }}_{0,q}^{a,b}}\;\;.$$

For the root of the channel (arm 0) we substitute $s_{0}^{0,0}$ from (7) in (9) and obtain that $x_{0,0}^{0,0}$ is a free parameter and in addition

$$\sigma_{0}=\alpha_{0,0}^{0,0}+{\hat{\gamma }}_{0,0}^{0,0}.$$

For the arm *r* which arises from node *m* of arm *q*, $dx_{r,0}^{q,m}/dt=0$ and then from model equations above we obtain

(10) $$x_{r,0}^{*q,m}=\frac{\delta_{r,q,m}^{a,b}}{\alpha_{r,0}^{q,m}+{\hat{\gamma }}_{r,0}^{q,m}}x_{q,m}^{*c,d}.$$

In principle the solution of Equations (8) is

(11) $$\begin{array}{ccc} x_{q,i}^{a,b} & = & x_{q,i}^{*a,b}+\sum_{j=0}^{i}b_{q,i,j}^{a,b}\exp\left[-\left(\alpha_{q,j}^{a,b}+j\beta_{q,j}^{a,b}+{\hat{\gamma }}_{q,j}^{a,b}\right)t\right],\\ & & i=1,2,\ldots , \end{array}$$

where $x_{q,i}^{*a,b}$ is stationary part of solution. We note that because of the non-negative values of the parameters α, β, and $\hat{\gamma }$, $x_{q,i}^{a,b}$ converges to $x_{q,i}^{*a,b}$ with increasing time.

For $x_{q,i}^{*a,b}$ one obtains the relationship (just set $dx_{q,i}^{a,b}/dt=0$ in Equations (8))

(12) $$x_{q,i}^{*a,b}=\frac{\alpha_{q,i-1}^{a,b}+\left(i-1\right)\beta_{q,i-1}^{a,b}}{\alpha_{q,i}^{a,b}+i\beta_{q,i}^{a,b}+{\hat{\gamma }}_{q,i}^{a,b}}x_{q,i-1}^{*a,b},\;i=1,2,\ldots .$$

The corresponding relationships for coefficients $b_{ij}^{q}$ are (i=1,…):

(13) $$\begin{array}{c} b_{q,i,j}^{a,b}=\frac{\alpha_{q,i-1}^{a,b}+\left(i-1\right)\beta_{q,i-1}^{a,b}}{\left(\alpha_{q,i}^{a,b}-\alpha_{q,i}^{a,b}\right)+\left(i\beta_{q,i}^{a,b}-j\beta_{q,j}^{a,b}\right)+\left({\hat{\gamma }}_{q,i}^{a,b}-{\hat{\gamma }}_{q,j}^{a,b}\right)}b_{q,i-1,j}^{a,b},\\ j=0,1,\ldots ,i-1, \end{array}$$

From Equation (12) one obtains (i=1,2,…)

(14) $$x_{q,i}^{*a,b}=\frac{\prod_{j=0}^{i-1}\left[\alpha_{q,i-j-1}^{a,b}+\left(i-j-1\right)\beta_{q,i-j-1}^{a,b}\right]}{\prod_{j=0}^{i-1}\left[\alpha_{q,i-j}^{a,b}+\left(i-j\right)\beta_{q,i-j}^{a,b}+{\hat{\gamma }}_{q,i-j}^{a,b}\right]}x_{q,0}^{*a,b}$$

The total amount of substance in the nodes of arm *q* is

(15) $$\begin{array}{c} x_{q}^{*a,b}=\sum_{i=0}^{\infty }x_{q,i}^{*a,b}=x_{q,0}^{*a,b}+\sum_{i=1}^{\infty }\frac{\prod_{j=0}^{i-1}\left[\alpha_{q,i-j-1}^{a,b}+\left(i-j-1\right)\beta_{q,i-j-1}^{a,b}\right]}{\prod_{j=0}^{i-1}\left[\alpha_{q,i-j}^{a,b}+\left(i-j\right)\beta_{q,i-j}^{a,b}+{\hat{\gamma }}_{q,i-j}^{a,b}\right]}x_{q,0}^{*a,b}. \end{array}$$

The form of corresponding probability distribution $y_{q,i}^{*a,b}=x_{q,i}^{*a,b}/x_{q}^{*a,b}$ is

(16) $$\begin{array}{c} y_{q,0}^{*a,b}=\frac{1}{1+\sum_{i=1}^{\infty }\frac{\prod_{j=0}^{i-1}\left[\alpha_{q,i-j-1}^{a,b}+\left(i-j-1\right)\beta_{q,i-j-1}^{a,b}\right]}{\prod_{j=0}^{i-1}\left[\alpha_{q,i-j}^{a,b}+\left(i-j\right)\beta_{q,i-j}^{a,b}+\gamma_{q,i-j}^{*a,b}\right]}}\\ y_{q,i}^{*a,b}=\frac{\frac{\prod_{j=0}^{i-1}\left[\alpha_{q,i-j-1}^{a,b}+\left(i-j-1\right)\beta_{q,i-j-1}^{a,b}\right]}{\prod_{j=0}^{i-1}\left[\alpha_{q,i-j}^{a,b}+\left(i-j\right)\beta_{q,i-j}^{a,b}+{\hat{\gamma }}_{q,i-j}^{a,b}\right]}}{1+\sum_{i=1}^{\infty }\frac{\prod_{j=0}^{i-1}\left[\alpha_{q,i-j-1}^{a,b}+\left(i-j-1\right)\beta_{q,i-j-1}^{a,b}\right]}{\prod_{j=0}^{i-1}\left[\alpha_{q,i-j}^{a,b}+\left(i-j\right)\beta_{q,i-j}^{a,b}+{\hat{\gamma }}_{q,i-j}^{a,b}\right]}}\\ i=1,2,\ldots \end{array}$$

We can write probability distribution connected to distribution of substance in a channel containing *M* arms (M=1,2,…). The total amount of substance in the arms of the channel is

(17) $$\begin{array}{c} x^{*}=\sum_{q=0}^{M}x_{q,0}^{*a,b}\left[1+\sum_{i=1}^{\infty }\frac{\prod_{j=0}^{i-1}\left[\alpha_{q,i-j-1}^{a,b}+\left(i-j-1\right)\beta_{q,i-j-1}^{a,b}\right]}{\prod_{j=0}^{i-1}\left[\alpha_{q,i-j}^{a,b}+\left(i-j\right)\beta_{q,i-j}^{a,b}+{\hat{\gamma }}_{q,i-j}^{a,b}\right]}\right]. \end{array}$$

The probability distribution connected to entire channel is as follows. For the 0-th node of *p*-th arm of the channel

(18) $$\begin{array}{c} z_{p,0}^{*a_{p},b_{p}}=\frac{x_{p,0}^{*,a_{p},b_{p}}}{\sum_{q=0}^{M}x_{q,0}^{*a,b}\left[1+\sum_{i=1}^{\infty }\frac{\prod_{j=0}^{i-1}\left[\alpha_{q,i-j-1}^{a,b}+\left(i-j-1\right)\beta_{q,i-j-1}^{a,b}\right]}{\prod_{j=0}^{i-1}\left[\alpha_{q,i-j}^{a,b}+\left(i-j\right)\beta_{q,i-j}^{a,b}+{\hat{\gamma }}_{q,i-j}^{a,b}\right]}\right]}, \end{array}$$

and for the *i*-th node of the *p*-th arm of the channel (i=1,2,,…)

(19) $$\begin{array}{c} z_{p,i}^{*a_{p},b_{p}}=\frac{\frac{\prod_{j=0}^{i-1}\left[\alpha_{p,i-j-1}^{a,b}+\left(i-j-1\right)\beta_{p,i-j-1}^{a,b}\right]}{\prod_{j=0}^{i-1}\left[\alpha_{p,i-j}^{a,b}+\left(i-j\right)\beta_{p,i-j}^{a,b}+{\hat{\gamma }}_{p,i-j}^{a,b}\right]}x_{p,0}^{*a,b}}{\sum_{q=0}^{M}x_{q,0}^{*a,b}\left[1+\sum_{i=1}^{\infty }\frac{\prod_{j=0}^{i-1}\left[\alpha_{q,i-j-1}^{a,b}+\left(i-j-1\right)\beta_{q,i-j-1}^{a,b}\right]}{\prod_{j=0}^{i-1}\left[\alpha_{q,i-j}^{a,b}+\left(i-j\right)\beta_{q,i-j}^{a,b}+{\hat{\gamma }}_{q,i-j}^{a,b}\right]}\right]} \end{array}$$

To the best of our knowledge the distributions presented by (16), (18) and (19) have been not discussed up to now outside our research group, in other words, they are new probability distributions. We note that for case of channel containing just one arm the obtained probability distributions reduce to distribution discussed in the Appendix of [23]. This distribution is connected to the long-tail distribution of Waring (Edward Waring was the 6-th Lucasian professor in mathematics at University of Cambridge).

### 2.2. Theory for the Case of Channel Consisting of Arms Containing Finite Number of Nodes

We consider a channel containing main arm labeled by 0 and number *M* of other arms. The arm *q* of this channel has finite number of $N_{q}+1$ nodes (labeled from 0 to $N_{q}$). The mathematical model for this case consists of a system of equations which contains an equation for 0-th node, equations for nodes $1,\ldots ,N_{q-1}$ and equation for node $N_{q}$. The model system of equations for node 0 and for nodes $1,\ldots ,N_{q-1}$ of *q*-th arm of the channel is (notations are the same as in the previous section)

(20) $$\begin{array}{ccc} \frac{dx_{q,0}^{a,b}}{dt} & = & s_{q}^{a,b}-\alpha_{q,0}^{a,b}x_{q,0}^{a,b}-{\hat{\gamma }}_{q,0}^{a,b}x_{q,0}^{a,b}, \end{array}$$

(21) $$\begin{array}{ccc} \frac{dx_{q,i}^{a,b}}{dt} & = & \left[\alpha_{q,i-1}^{a,b}+\left(i-1\right)\beta_{q,i-1}^{a,b}\right]x_{q,i-1}^{a,b}-\left(\alpha_{q,i}^{a,b}+i\beta_{q,i}^{a,b}+{\hat{\gamma }}_{q,i}^{a,b}\right)x_{q,i}^{a,b};\\ & & i=1,2,\ldots ,N_{q}-1. \end{array}$$

For node $N_{q}$ of *q*-th arm there is no outflow to next mode of the arm (as node $N_{q}$ is the last node of *q*-th arm). Thus the equation for motion of substance for this node is

(22) $$\begin{array}{ccc} \frac{dx_{q,N}^{a,b}}{dt} & = & \left[\alpha_{q,N_{q}-1}^{a,b}+\left(N_{q}-1\right)\beta_{q,N_{q}-1}^{a,b}\right]x_{q,N_{q}-1}^{a,b}-{\hat{\gamma }}_{q,N_{q}}^{a,b}x_{q,N_{q}}^{a,b}. \end{array}$$

We discuss the case of stationary motion of substance through arms of studied channel. Then $dx_{0}^{q}/dt=0$ in (20). Hence

(23) $$x_{q,0}^{*a,b}=\frac{s_{q}^{a,b}}{\alpha_{0,q}^{a,b}+{\hat{\gamma }}_{0,q}^{a,b}}\;\;.$$

For root of the channel (arm 0) we substitute $s_{0}^{0,0}$ from Equation (7) in Equation (23) and obtain that $x_{0,0}^{0,0}$ is a free parameter and in addition

$$\sigma_{0}=\alpha_{0,0}^{0,0}+{\hat{\gamma }}_{0,0}^{0,0}.$$

For arm *r* which arises from node *m* of arm *q*, $dx_{r,0}^{q,m}/dt=0$ and then from the model equations above we obtain

(24) $$x_{r,0}^{*q,m}=\frac{\delta_{r,q,m}^{a,b}}{\alpha_{r,0}^{q,m}+{\hat{\gamma }}_{r,0}^{q,m}}x_{q,m}^{*c,d}.$$

For $x_{q,i}^{*a,b}$ we obtain the relationship (just set $dx_{q,i}^{a,b}/dt=0$ in (21))

(25) $$x_{q,i}^{*a,b}=\frac{\alpha_{q,i-1}^{a,b}+\left(i-1\right)\beta_{q,i-1}^{a,b}}{\alpha_{q,i}^{a,b}+i\beta_{q,i}^{a,b}+{\hat{\gamma }}_{q,i}^{a,b}}x_{q,i-1}^{*a,b},\;i=1,2,\ldots ,N_{q}-1;$$

In order to calculate $x_{q,N_{q}}^{*a,b}$ we use (22). The result is

(26) $$x_{q,N_{q}}^{*a,b}=\frac{\alpha_{q,N_{q}-1}^{a,b}+\left(N_{q}-1\right)\beta_{q,N_{q}-1}^{a,b}}{{\hat{\gamma }}_{q,N_{q}}^{a,b}}x_{q,N_{q}-1}^{*a,b}.$$

What follows from (25) is

(27) $$\begin{array}{c} x_{q,i}^{*a,b}=\frac{\prod_{j=0}^{i-1}\left[\alpha_{q,i-j-1}^{a,b}+\left(i-j-1\right)\beta_{q,i-j-1}^{a,b}\right]}{\prod_{j=0}^{i-1}\left[\alpha_{q,i-j}^{a,b}+\left(i-j\right)\beta_{q,i-j}^{a,b}+{\hat{\gamma }}_{q,i-j}^{a,b}\right]}x_{q,0}^{*a,b},\;\;i=1,\ldots ,N_{q}-1. \end{array}$$

And from (26) we obtain

(28) $$\begin{array}{c} x_{q,N_{q}}^{*a,b}=x_{q,0}^{*a,b}\;\frac{\alpha_{q,N_{q}-1}^{a,b}+\left(N_{q}-1\right)\beta_{q,N_{q}-1}^{a,b}}{{\hat{\gamma }}_{q,N_{q}}^{a,b}}\times \\ \frac{\prod_{j=0}^{N_{q}-2}\left[\alpha_{q,N_{q}-j-2}^{a,b}+\left(N_{q}-j-2\right)\beta_{q,N_{q}-j-2}^{a,b}\right]}{\prod_{j=0}^{N_{q}-2}\left[\alpha_{q,N_{q}-j-1}^{a,b}+\left(N_{q}-j-1\right)\beta_{q,N_{q}-j-1}^{a,b}+{\hat{\gamma }}_{q,N_{q}-j-1}^{a,b}\right]}. \end{array}$$

The total amount of the substance in *q*-th arm of channel is

(29) $$\begin{array}{c} x_{q}^{*,a,b}=x_{q,0}^{*a,b}+\sum_{i=1}^{N_{q}-1}x_{q,i}^{*,a,b}+x_{q,N}^{*,a,b}=x_{q,0}^{*a,b}A_{q} \end{array}$$

where *A* is given by relationship

(30) $$\begin{array}{c} A_{q}=1+\sum_{i=1}^{N_{q}-1}\frac{\prod_{j=0}^{i-1}\left[\alpha_{q,i-j-1}^{a,b}+\left(i-j-1\right)\beta_{q,i-j-1}^{a,b}\right]}{\prod_{j=0}^{i-1}\left[\alpha_{q,i-j}^{a,b}+\left(i-j\right)\beta_{q,i-j}^{a,b}+{\hat{\gamma }}_{q,i-j}^{a,b}\right]}+\frac{\alpha_{q,N_{q}-1}^{a,b}+\left(N_{q}-1\right)\beta_{q,N_{q}-1}^{a,b}}{{\hat{\gamma }}_{q,N_{q}}^{a,b}}\times \\ \frac{\prod_{j=0}^{N_{q}-2}\left[\alpha_{q,N_{q}-j-2}^{a,b}+\left(N_{q}-j-2\right)\beta_{q,N_{q}-j-2}^{a,b}\right]}{\prod_{j=0}^{N_{q}-2}\left[\alpha_{q,N_{q}-j-1}^{a,b}+\left(N_{q}-j-1\right)\beta_{q,N_{q}-j-1}^{a,b}+{\hat{\gamma }}_{q,N_{q}-j-1}^{a,b}\right]}. \end{array}$$

The distribution of substance in nodes of *q*-th arm of channel is

(31) $$\begin{array}{c} y_{q,0}^{*a,b}=\frac{1}{A_{q}}, \end{array}$$

(32) $$\begin{array}{c} y_{q,i}^{*a,b}=\frac{B_{q,i}}{A_{q}}, \end{array}$$

where

(33) $$\begin{array}{c} B_{q,i}=\frac{\prod_{j=0}^{i-1}\left[\alpha_{q,i-j-1}^{a,b}+\left(i-j-1\right)\beta_{q,i-j-1}^{a,b}\right]}{\prod_{j=0}^{i-1}\left[\alpha_{q,i-j}^{a,b}+\left(i-j\right)\beta_{q,i-j}^{a,b}+{\hat{\gamma }}_{q,i-j}^{a,b}\right]}\;\;i=1,\ldots ,N_{q}-1, \end{array}$$

(34) $$\begin{array}{c} y_{q,N_{q}}^{*a,b}=\frac{B_{q,N_{q}}}{A_{q}}, \end{array}$$

where $B_{q,N_{q}}$ is given by the relationship

(35) $$\begin{array}{c} B_{q,N_{q}}=\frac{\alpha_{q,N_{q}-1}^{a,b}+\left(N_{q}-1\right)\beta_{q,N_{q}-1}^{a,b}}{{\hat{\gamma }}_{q,N_{q}}^{a,b}}\times \\ \frac{\prod_{j=0}^{N_{q}-2}\left[\alpha_{q,N_{q}-j-2}^{a,b}+\left(N_{q}-j-2\right)\beta_{q,N_{q}-j-2}^{a,b}\right]}{\prod_{j=0}^{N_{q}-2}\left[\alpha_{q,N_{q}-j-1}^{a,b}+\left(N_{q}-j-1\right)\beta_{q,N_{q}-j-1}^{a,b}+{\hat{\gamma }}_{q,N_{q}-j-1}^{a,b}\right]}. \end{array}$$

We can write also the probability distribution of substance for entire channel, in other words, for *M* branches of channel. The total amount of substance in this case is

(36) $$\begin{array}{c} x^{*}=\sum_{q=0}^{M}x_{q,0}^{*a_{q},b_{q}}A_{q}. \end{array}$$

Distribution of substance in entry nodes of arms (p=0,…,M) of the channel is:

(37) $$\begin{array}{c} z_{p,0}^{*a_{p},b_{p}}=\frac{x_{p,0}^{*a_{p},b_{p}}}{\sum_{q=0}^{M}x_{q,0}^{*a_{q},b_{q}}A_{q}}. \end{array}$$

Distribution of substance in interior nodes of the arms is:

(38) $$\begin{array}{c} z_{p,i}^{*a_{p},b_{p}}=\frac{x_{p,i}^{*,a_{p},b_{p}}}{\sum_{q=0}^{M}x_{q,0}^{*a_{q},b_{q}}A_{q}}=\frac{B_{p,i}x_{p,0}^{*a_{p},b_{p}}}{\sum_{q=0}^{M}x_{q,0}^{*a_{q},b_{q}}A_{q}},\;\;i=1,\ldots ,N_{q-1}. \end{array}$$

Distribution of substance in last nodes of arms of the channel is:

(39) $$\begin{array}{c} z_{p,N_{p}}^{*a_{p},b_{p}}=\frac{x_{p,N_{p}}^{*a_{p},b_{p}}}{\sum_{q=0}^{M}x_{q,0}^{*a_{q},b_{q}}A_{q}}=\frac{B_{p,N_{p}}x_{p,0}^{*a_{p},b_{p}}}{\sum_{q=0}^{M}x_{q,0}^{*a_{q},b_{q}}A_{q}}. \end{array}$$

To the best of our knowledge the distributions presented by (31)–(34) and (37)–(39) are not discussed up to now outside our research group. In other words, these are new probability distributions. The obtained distributions are interesting for the practice as they are connected to class of channels containing finite number of arms and in addition each arm contains finite number of nodes.

## 3. Information Measures Connected to Obtained Probability Distributions

We can calculate various quantities connected to the obtained distributions. Below we consider an example related to an information problem. Let us consider flow of substance in the channel of network studied above. Each node of the channel is numbered and we can consider these nodes as letters of an alphabet. Let some kind of event happens in any of the nodes and let probability of occurrence of this event be proportional of amount of substance in corresponding node. Thus probability of occurrence of event in a node of channel will be equal to the probability from the corresponding probability distribution obtained above in the text. The channel (the source) will generate events with corresponding probability and we can calculate measure of information and Shannon measure of information for these sequences.

The information measure connected to an event with probability *p* is

(40) $$I_{p}=-\log\left(p\right),$$

and Shannon information measure (average information we get from a symbol in a stream) connected to probability distribution $P=(p_{0},\ldots ,p_{N})$ is

(41) $$H\left(P\right)=-\sum_{i=0}^{N}p_{i}\log\left(p_{i}\right)$$

Let us consider the distribution $P^{*}$ of substance in *q*-th arm of the channel given by (31)–(35). The information connected to event with probability $p_{i}$ from *i*-th node of this arm is

(42) $$\begin{array}{ccc} I_{p_{0}} & = & \log(A_{q})\\ I_{p_{i}} & = & \log\left(A_{q}\right)-\log\left(B_{q,i}\right),\;\;i=1,\ldots ,N_{q}-1\\ I_{p_{N}} & = & \log\left(A_{q}\right)-\log\left(B_{q,N_{q}}\right). \end{array}$$

The Shannon information measure connected to distribution $P^{*}$ is

(43) $$\begin{array}{c} H\left(P^{*}\right)=\frac{\log(A_{q})}{A_{q}}-\sum_{i=1}^{N_{q}-1}\frac{B_{q,i}}{A_{q}}\log\left(\frac{B_{q,i}}{A_{q}}\right)-\frac{B_{q,N_{q}}}{A_{q}}\log\left(\frac{B_{q,N_{q}}}{A_{q}}\right). \end{array}$$

Let us consider now a very simple case: a channel containing single arm that has just three nodes. Below we write information measures for the nodes as well as Shannon information measure for this channel. We shall omit the indices *a*, *b* and *q*. We note that for this case $N_{q}=N=2$ as labels of nodes of the arm are 0, 1 and 2. The probabilities connected to three nodes of studied channel are

(44) $$\begin{array}{ccc} p_{0} & = & \frac{1}{1+\frac{\alpha_{0}}{\alpha_{1}+\beta_{1}+{\hat{\gamma }}_{1}}\left(1+\frac{\alpha_{1}+\beta_{1}}{{\hat{\gamma }}_{2}}\right)},\\ p_{1} & = & \frac{\frac{\alpha_{0}}{\alpha_{1}+\beta_{1}+{\hat{\gamma }}_{1}}}{1+\frac{\alpha_{0}}{\alpha_{1}+\beta_{1}+{\hat{\gamma }}_{1}}\left(1+\frac{\alpha_{1}+\beta_{1}}{{\hat{\gamma }}_{2}}\right)},\\ p_{2} & = & \frac{\frac{\alpha_{1}+\beta_{1}}{{\hat{\gamma }}_{2}}\frac{\alpha_{0}}{\alpha_{1}+\beta_{1}+{\hat{\gamma }}_{1}}}{1+\frac{\alpha_{0}}{\alpha_{1}+\beta_{1}+{\hat{\gamma }}_{1}}\left(1+\frac{\alpha_{1}+\beta_{1}}{{\hat{\gamma }}_{2}}\right)}. \end{array}$$

The parameters in (44) account for following processes

- $\alpha_{0}$ ($0<\alpha_{0}<1$): flow between first and second node,
- $\alpha_{1}$ ($0<\alpha_{1}<1$): flow between second and third node,
- $\beta_{1}$ ($0<\beta_{1}<1-\alpha_{1}$): preference for the third node,
- ${\hat{\gamma }}_{1}$ ($0\leq {\hat{\gamma }}_{1}<1-\alpha_{1}-\beta_{1}$): leakage from the second node,
- ${\hat{\gamma }}_{2}$ ($0<{\hat{\gamma }}_{2}\leq 1$): leakage from the third node.

The information measures connected to nodes are

(45) $$\begin{array}{ccc} I_{p_{0}} & = & \log\left[1+\frac{\alpha_{0}}{\alpha_{1}+\beta_{1}+{\hat{\gamma }}_{1}}\left(1+\frac{\alpha_{1}+\beta_{1}}{{\hat{\gamma }}_{2}}\right)\right]\\ I_{p_{1}} & = & \log\left[1+\frac{\alpha_{0}}{\alpha_{1}+\beta_{1}+{\hat{\gamma }}_{1}}\left(1+\frac{\alpha_{1}+\beta_{1}}{{\hat{\gamma }}_{2}}\right)\right]-\log\left(\frac{\alpha_{0}}{\alpha_{1}+\beta_{1}+{\hat{\gamma }}_{1}}\right)\\ I_{p_{2}} & = & \log\left[1+\frac{\alpha_{0}}{\alpha_{1}+\beta_{1}+{\hat{\gamma }}_{1}}\left(1+\frac{\alpha_{1}+\beta_{1}}{{\hat{\gamma }}_{2}}\right)\right]-\\ & & \log\left(\frac{\alpha_{1}+\beta_{1}}{{\hat{\gamma }}_{2}}\frac{\alpha_{0}}{\alpha_{1}+\beta_{1}+{\hat{\gamma }}_{1}}\right). \end{array}$$

The corresponding Shannon information measure is

(46) $$\begin{array}{ccc} H & = & \frac{1}{\left[1+\frac{\alpha_{0}}{\alpha_{1}+\beta_{1}+{\hat{\gamma }}_{1}}\left(1+\frac{\alpha_{1}+\beta_{1}}{{\hat{\gamma }}_{2}}\right)\right]}\log\left[1+\frac{\alpha_{0}}{\alpha_{1}+\beta_{1}+{\hat{\gamma }}_{1}}\left(1+\frac{\alpha_{1}+\beta_{1}}{{\hat{\gamma }}_{2}}\right)\right]+\\ & & \frac{\left(\frac{\alpha_{0}}{\alpha_{1}+\beta_{1}+{\hat{\gamma }}_{1}}\right)}{\left[1+\frac{\alpha_{0}}{\alpha_{1}+\beta_{1}+{\hat{\gamma }}_{1}}\left(1+\frac{\alpha_{1}+\beta_{1}}{{\hat{\gamma }}_{2}}\right)\right]}\left\{\log\left[1+\frac{\alpha_{0}}{\alpha_{1}+\beta_{1}+{\hat{\gamma }}_{1}}\left(1+\frac{\alpha_{1}+\beta_{1}}{{\hat{\gamma }}_{2}}\right)\right]-\right.\\ & & \left.\log\left(\frac{\alpha_{0}}{\alpha_{1}+\beta_{1}+{\hat{\gamma }}_{1}}\right)\right\}+\frac{\left(\frac{\alpha_{1}+\beta_{1}}{{\hat{\gamma }}_{2}}\frac{\alpha_{0}}{\alpha_{1}+\beta_{1}+{\hat{\gamma }}_{1}}\right)}{\left[1+\frac{\alpha_{0}}{\alpha_{1}+\beta_{1}+{\hat{\gamma }}_{1}}\left(1+\frac{\alpha_{1}+\beta_{1}}{{\hat{\gamma }}_{2}}\right)\right]}\times \\ & & \left\{\log\left[1+\frac{\alpha_{0}}{\alpha_{1}+\beta_{1}+{\hat{\gamma }}_{1}}\left(1+\frac{\alpha_{1}+\beta_{1}}{{\hat{\gamma }}_{2}}\right)\right]-\right.\left.\log\left(\frac{\alpha_{1}+\beta_{1}}{{\hat{\gamma }}_{2}}\frac{\alpha_{0}}{\alpha_{1}+\beta_{1}+{\hat{\gamma }}_{1}}\right)\right\}. \end{array}$$

**Figure 3:**
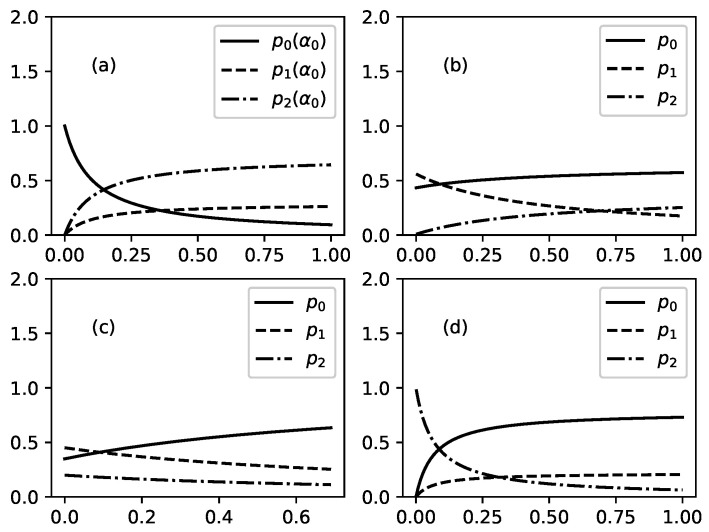
Probabilities $p_{0}$ (solid lines), $p_{1}$ (dashed lines), and $p_{2}$ (dot-dashed lines) as functions of selected parameter of the problem when all other parameters are fixed. (**a**): $p_{0}\left(\alpha_{0}\right)$, $p_{1}\left(\alpha_{0}\right)$; $p_{2}\left(\alpha_{0}\right)$ for fixed values of other parameters as follows: $\alpha_{1}=0.04$, $\beta_{1}=0.28$, ${\hat{\gamma }}_{1}=0.04$, ${\hat{\gamma }}_{2}=0.13$. (**b**): $p_{0}\left(\alpha_{1}\right)$, $p_{1}\left(\alpha_{1}\right)$; $p_{2}\left(\alpha_{1}\right)$ for fixed values of other parameters as follows: $\alpha_{0}=0.4$, $\beta_{1}=0.01$, ${\hat{\gamma }}_{1}=0.3$, ${\hat{\gamma }}_{2}=0.7$. (**c**): $p_{0}\left({\hat{\gamma }}_{1}\right)$, $p_{1}\left({\hat{\gamma }}_{1}\right)$; $p_{2}\left({\hat{\gamma }}_{1}\right)$ for fixed values of other parameters as follows: $\alpha_{0}=0.4$, $\alpha_{1}=0.3$, $\beta_{1}=0.01$, ${\hat{\gamma }}_{2}=0.7$. (**d**): $p_{0}\left({\hat{\gamma }}_{2}\right)$, $p_{1}\left({\hat{\gamma }}_{2}\right)$; $p_{2}\left({\hat{\gamma }}_{2}\right)$ for fixed values of other parameters as follows: $\alpha_{0}=0.2$, $\alpha_{1}=0.3$, $\beta_{1}=0.01$, ${\hat{\gamma }}_{1}=0.4$.

**Figure 4:**
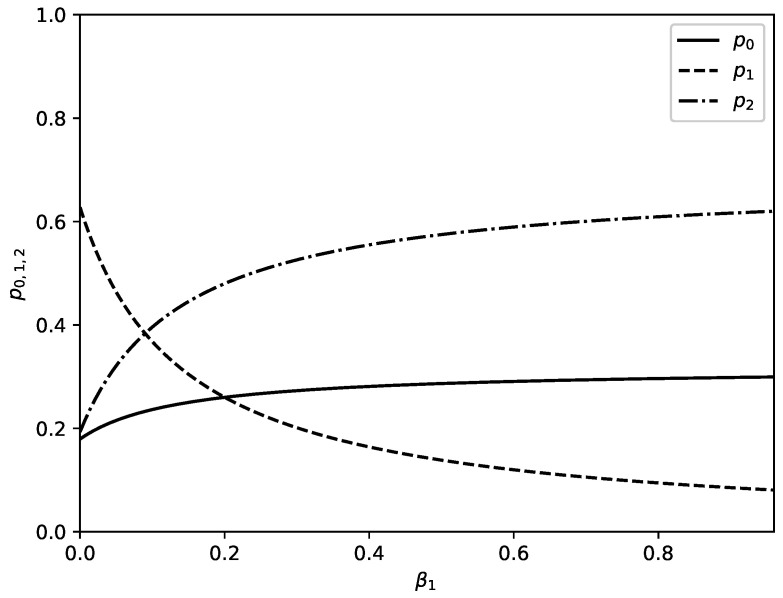
Probabilities $p_{0}\left(\beta_{1}\right)$ (solid lines), $p_{1}\left(\beta_{1}\right)$ (dashed lines), and $p_{2}\left(\beta_{1}\right)$ (dot-dashed lines) as functions of $\beta_{1}$ when all other parameters are fixed as follows: $\alpha_{0}=0.28$, $\alpha_{1}=0.04$, ${\hat{\gamma }}_{1}=0.04$, ${\hat{\gamma }}_{2}=0.13$.

**Figure 5:**
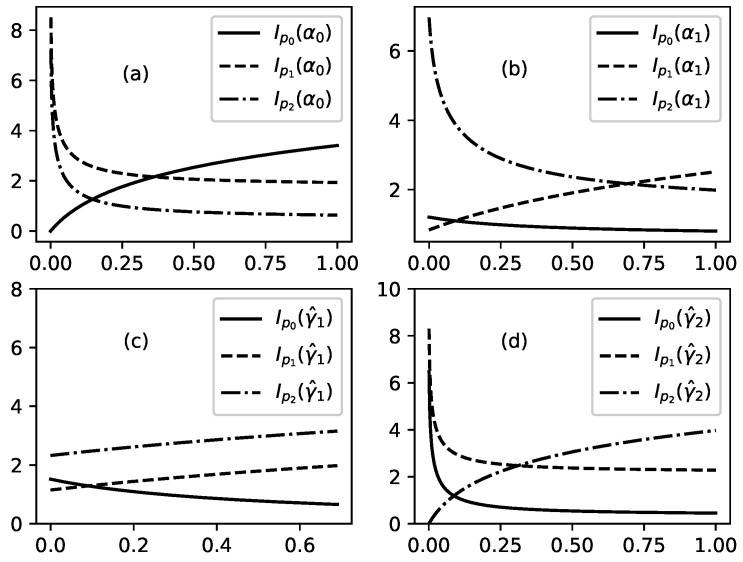
Information measures $I_{p_{0}}$ (solid lines), $I_{p_{1}}$ (dashed lines), and $I_{p_{2}}$ (dot-dashed lines) as functions of selected parameter of the problem when all other parameters are fixed. (**a**): Dependence of $I_{p_{0}}$, $I_{p_{1}}$; $I_{p_{2}}$ on $\alpha_{0}$ for fixed values of other parameters as follows: $\alpha_{1}=0.04$, $\beta_{1}=0.28$, ${\hat{\gamma }}_{1}=0.04$, ${\hat{\gamma }}_{2}=0.13$. (**b**): Dependence of $I_{p_{0}}$, $I_{p_{1}}$; $I_{p_{2}}$ on $\alpha_{1}$ for fixed values of other parameters as follows: $\alpha_{0}=0.4$, $\beta_{1}=0.01$, ${\hat{\gamma }}_{1}=0.3$, ${\hat{\gamma }}_{2}=0.7$. (**c**): Dependence of $I_{p_{0}}$, $I_{p_{1}}$; $I_{p_{2}}$ on ${\hat{\gamma }}_{1}$ for fixed values of other parameters as follows: $\alpha_{0}=0.4$, $\alpha_{1}=0.3$, $\beta_{1}=0.01$, ${\hat{\gamma }}_{2}=0.7$. (**d**): Dependence of $I_{p_{0}}$, $I_{p_{1}}$; $I_{p_{2}}$ on ${\hat{\gamma }}_{2}$ for fixed values of other parameters as follows: $\alpha_{0}=0.2$, $\alpha_{1}=0.3$, $\beta_{1}=0.01$, ${\hat{\gamma }}_{1}=0.4$.

**Figure 6:**
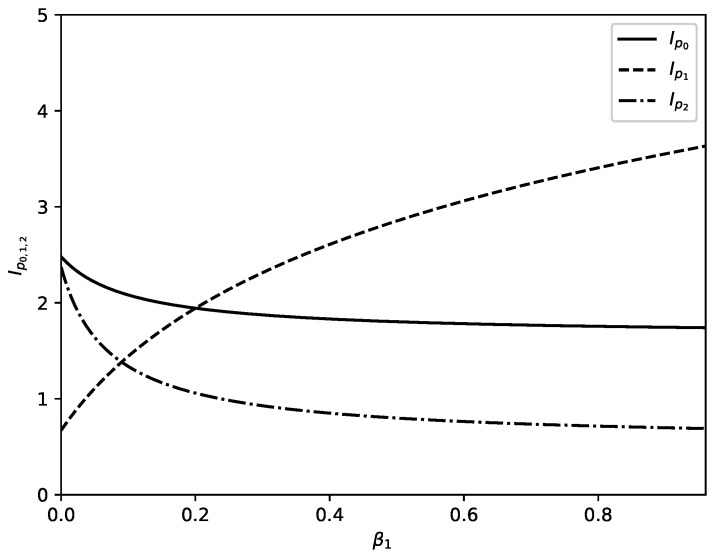
$I_{p_{0}}$ (solid lines), $I_{p_{1}}$ (dashed lines), and $I_{p_{2}}$ (dot-dashed lines) as functions of $\beta_{1}$ when all other parameters are fixed as follows: $\alpha_{0}=0.28$, $\alpha_{1}=0.04$, ${\hat{\gamma }}_{1}=0.04$, ${\hat{\gamma }}_{2}=0.13$.

**Figure 7:**
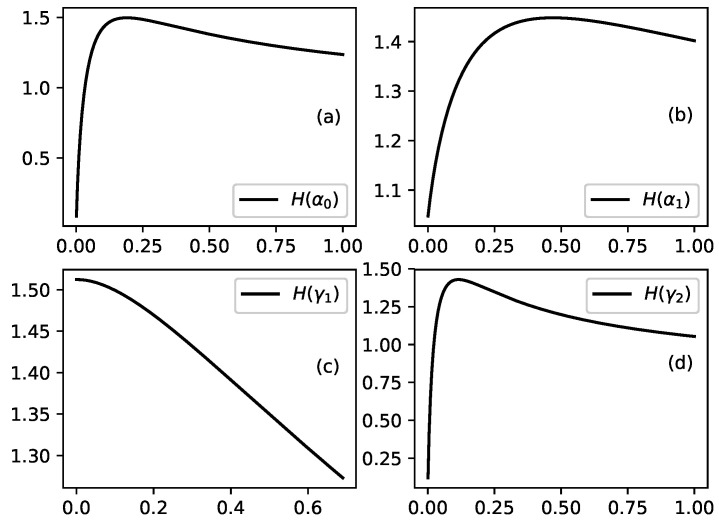
Shannon information measure as function of selected parameters when the other parameters of probability distribution are fixed. (**a**): $H(\alpha_{0})$ for fixed values of other parameters as follows: $\alpha_{1}=0.04$, $\beta_{1}=0.28$, ${\hat{\gamma }}_{1}=0.04$, ${\hat{\gamma }}_{2}=0.13$. (**b**): $H(\alpha_{1})$ for fixed values of other parameters as follows: $\alpha_{0}=0.4$, $\beta_{1}=0.01$, ${\hat{\gamma }}_{1}=0.3$, ${\hat{\gamma }}_{2}=0.7$. (**c**): $H({\hat{\gamma }}_{1}$) for fixed values of other parameters as follows: $\alpha_{0}=0.4$, $\alpha_{1}=0.3$, $\beta_{1}=0.01$, ${\hat{\gamma }}_{2}=0.7$. (**d**): $H({\hat{\gamma }}_{2})$ for fixed values of other parameters as follows: $\alpha_{0}=0.2$, $\alpha_{1}=0.3$, $\beta_{1}=0.01$, ${\hat{\gamma }}_{1}=0.4$.

Several illustrations for the dependence of $p_{0,1,2}$, $I_{p_{0},p_{1},p_{2}}$ and *H* on parameters of problem are presented in Figure 3, Figure 4, Figure 5, Figure 6 and Figure 7.

Figure 3 shows influence of the parameters of problem on probabilities $p_{0,1,2}$ (connected to stationary distribution of substance in the three nodes of studied channel). Figure 3a shows the influence of $\alpha_{0}$ on $p_{0,1,2}$ when other parameters are fixed. $\alpha_{0}$ is a parameter which regulates the outflow of substance from node 0 to node 1 of channel. For this case the probabilities can be written as follows:

$$p_{0}=\frac{1}{1+c_{0}\alpha_{0}};\;\;\;p_{1}=\frac{c_{1}\alpha_{0}}{1+c_{0}\alpha_{0}};\;\;\;p_{2}=\frac{c_{2}\alpha_{0}}{1+c_{0}\alpha_{0}},$$

where $c_{0},c_{1},c_{2}$ are appropriate constants which values depend on values of fixed parameters.

With increasing outflow from the node 0, $p_{0}$ decreases and $p_{1}$ and $p_{2}$ increase. This is connected to redistribution of percentage of total substance which is presented in any of the three nodes: percentage of the substance in node 0 decreases because of the increased outflow and this leads to increase of percentage of substance in other two nodes.

Figure 3b shows influence of increasing value of parameter $\alpha_{1}$ on the probabilities $p_{0,1,2}$. Parameter $\alpha_{1}$ accounts for the outflow of substance from node 1 to node 2 of studied channel. Increase of the value of $\alpha_{1}$ leads to decrease of percentage of substance in node 2 and to increase of percentage of substance in node 2. Interesting is what happens in node 0. For fixed values of parameters as in Figure 3b the percentage of substance in node 0 increases but for other values of these parameters percentage of substance in node 0 can decrease.

Figure 3c shows influence of increasing value of the parameter ${\hat{\gamma }}_{1}$ on percentages of substance in the three nodes of channel. As there are no branches in studied channel then ${\hat{\gamma }}_{1}=\gamma_{1}$. Parameter $\gamma_{1}$ accounts for the leakage from node 1 of channel. The increase of this leakage leads to decrease of percentage of substance in node 1 and in following node 2 at expense of percentage of substance in node 0 (node 0 is not affected by the leakage of substance in node 1 which position is after node 0).

Figure 3d shows influence of increasing value of the parameter ${\hat{\gamma }}_{2}$ on percentage of substance in nodes 0, 1, and 2. Parameter ${\hat{\gamma }}_{2}$ accounts for leakage of substance from node 2 of studied channel. There are no branches in studied channel and because of this ${\hat{\gamma }}_{2}=\gamma_{2}$. Increased value of ${\hat{\gamma }}_{2}$ (increased leakage from node 2) leads to decrease of percentage of substance in node 2 and to corresponding increase of substance in nodes 0 and 1.

Figure 4 shows influence of increasing value of the parameter $\beta_{1}$ on probabilities $p_{0,1,2}$. Parameter $\beta_{1}$ accounts for additional outflow of substance from node 1 to node 2 because of some extra reason (in the theory of migration this extra reason can be preference of migrants which prefer to migrate to country 2 instead to stay in country 1). Increased value of $\beta_{1}$ leads to decrease of percentage of substance in the node 1 and to increase of percentage of substance which is located in node 2. For the values of parameters as in Figure 4 there is an additional change: percentage of substance in node 0 increases too.

Figure 5 and Figure 6 show influence of changing values of parameters of problem on the values of information measures $I_{p_{0}}$, $I_{p_{1}}$, and $I_{p_{2}}$ for nodes of studied channel. Figure 5a shows changes in the information measures with increasing value of parameter $\alpha_{0}$ when values of all other parameters of problem are fixed. We observe that the value of $I_{p_{0}}$ increases with increasing value of $\alpha_{0}$ and values of $I_{p_{1}}$ and $I_{p_{2}}$ decrease with increasing value of $\alpha_{1}$. This is because of the redistribution of percentage of substance in nodes 0, 1 and 2 with increasing value of $\alpha_{0}$. Because of increasing outflow from node 0 the percentage of total substance located in this node decreases. The event associated with information measure *I* for node 0 becomes rare and occurrence of this event carries larger information. The percentage of substance in nodes 1 and 2 increases with increasing value of $\alpha_{0}$. The event associated with information measure *I* becomes more frequent and this leads to decreasing information associated with occurrence of this event in nodes 1 and 2. Similar is the situation in Figure 5b where increasing value of parameter $\alpha_{1}$ (accounting for outflow of substance from node 1 to node 2) leads to increasing percentage of substance in nodes 0 and 2 and decreasing percentage of substance in node 2. The information associated with occurrence of event of interest in node 2 increases and information associated with occurrence of event of interest in nodes 0 and 2 decreases.

Figure 5c shows influence of increasing value of the leakage parameter ${\hat{\gamma }}_{1}*$ (accounting for leakage from node 1) on information associated with occurrence of the event of interest in nodes 0, 1, and 2. Decreasing percentage of amount of substance in nodes 1 and 2 and increasing percentage of substance in node 0 lead to increasing information associated with occurrence of the event in nodes 1 and 2 and decreasing information associated with occurrence of event of interest in node 0. Situation connected to increasing value of the leakage parameter ${\hat{\gamma }}_{2}$ is shown in Figure 5d. This situation is similar to the situation from Figure 5c: increasing percentage of substance leads to decreasing value of information measure associated with occurrence of event of interest and decreasing percentage of substance leads to increasing value of information measure associated with occurrence of event of interest.

Figure 6 shows influence of increasing value of preference parameter $\beta_{1}$ on information measures $I_{p_{0}}$, $I_{p_{1}}$, and $I_{p_{2}}$. For corresponding fixed values of other parameters the increase of value of $\beta_{1}$ leads to increase of percentage of substance in nodes 0 and 2 and decrease of percentage of total substance located in node 2. The information associated with event of interest increases for events occurrence in node 2 and decreases for events occurrence in other two nodes.

Finally Figure 7 shows influence of increasing value of selected parameters on the Shannon information measure for entire channel. Shannon information measure *H* is the average information we get from a occurrence of event of interest in nodes of studied channel. Two kinds of behavior of Shannon information measure are shown in Figure 7. First of all increase of value of selected parameter (with fixed values of other parameters) can lead to a maximum of the value of Shannon information measure for some value of changing parameter as shown in Figure 7a,b,d. Second kind of behavior is shown in Figure 7c where increasing value of leakage parameter $\gamma_{1}$ leads to monotonous decrease of value of the Shannon information measure.

## 4. Discussion

The results obtained above allow us to discuss various kinds of probability distributions. The conventional probability distributions correspond to a channel which has a single arm. Such distributions have been discussed in our previous work [23,26,27,28]. These distributions can be connected to Waring distribution, Zipf distribution, Yule-Simon distribution, Binomial distribution, etc. We can study also other kinds of distributions. One example is the probability distribution connected to distribution of substance in a channel which has more than one arm. More complicated case is the probability distribution connected to distribution of substance in a part of the studied network that contains several channels for motion of substance. The most complicated kind of probability distribution is the probability distribution connected to distribution of substance in all nodes of studied network.

## 5. Concluding Remarks

We discuss above a model of directed motion of substance through a channel of a network. The study is devoted to the stationary regime of motion of substance through channel arms and main outcomes are obtained new distributions connected to distribution of substance in nodes of channel. The model is formulated in such a way that it can have broad range of applicability. For an example the model can be used for study of motion of substance in technological systems or for study of motion of resources in various networks (e.g., motion of goods in logistic networks). The model can be applied also for study of other systems such as channels of human migration. Let us finish the text by an interpretation of obtained results from point of view of migration flows. For the case of migration flow migrants move through countries which form a migration channel and some migrants obtain permission to stay in corresponding country (which corresponds to leakage phenomenon in our model). Figure 3 shows the influence of model parameters on distribution of migrants in corresponding channel. Especially interesting is the influence of increasing leakage parameter shown in Figure 3d. Increasing leakage $\gamma_{2}$ means an increase of number of migrants who obtain permission to stay in the third country of studied channel. This can lead to drop of percentage of migrants without permission to stay in this country at the expense of the percentage of migrants without permission to stay in the other two countries of channel. Figure 3c shows that increasing leakage in second country of studied channel affect the percentage of migrants in the third country of the channel. If migrants have preferences for the third country of studied channel then percentage of migrants in this country increases with increased preference mostly at expense of percentage of migrants in previous country of channel—Figure 4. Changes of parameters of the model affect information about events connected to flows of migrants (e.g., information about criminal events). With increasing permeability of borders between countries (for an example with increasing value of parameter $\alpha_{0}$) the amount of information connected to studied class of events increases in first country of studied channel and decreases in next two countries—Figure 5a. Opposite effect connected to increasing of leakage is shown in Figure 5c. Increasing value of preference parameter $\beta_{1}$ leads to increasing value of information connected with studied class of events in second country of the channel—Figure 6. Interesting is that the Shannon information measure connected to studied class of events can have maxima for selected values of model parameters—Figure 7.
